# Efficacy of home phototherapy versus inpatient phototherapy for neonatal hyperbilirubinemia: a systematic review and meta-analysis

**DOI:** 10.1186/s13052-024-01613-0

**Published:** 2024-03-04

**Authors:** Rui Li, Tingting Li, Xudong Yan, Jing Feng, Zhangbin Yu, Cheng Chen

**Affiliations:** 1 Division of Neonatology, Longgang District Maternity & Child Healthcare Hospital of Shenzhen City (Longgang Maternity and Child Institute of Shantou University Medical College), Shenzhen, China; 2 Department of Pediatrics Division of Neonatology, Shenzhen People’s Hospital (The Second Clinical Medical College, Jinan University, The First Affiliated Hospital, Southern University of Science and Technology), Shenzhen, China

**Keywords:** Home phototherapy, Neonatal hyperbilirubinemia, Efficacy, Meta-analysis

## Abstract

**Background:**

Home phototherapy (HPT) remains a contentious alternative to inpatient phototherapy (IPT) for neonatal hyperbilirubinemia. To guide evidence-based clinical decision-making, we conducted a meta-analysis of randomized clinical trials (RCTs) and cohort studies and assessed the comparative risks and benefits of HPT and IPT.

**Methods:**

PubMed, Embase, Web of Science, Cochrane Library, Chinese National Knowledge Infrastructure Database, Wanfang Database, Chinese Science and Technique Journals Database, ClinicalTrials.gov, and International Clinical Trial Registry Platform trial were searched from inception until June 2, 2023. We included RCTs and cohort studies and adhered to Preferred Reporting Items for Systematic Reviews and Meta-Analysis guidelines. Study quality was assessed with the Cochrane Collaboration Risk of Bias tool and the Newcastle–Ottawa scale. The outcome measures were phototherapy duration, daily bilirubin level reduction, exchange transfusion, hospital readmission, parental stress scale, and complications. We used fixed- or random-effects meta-analysis models, assessed heterogeneity (*I*^*2*^), conducted subgroup analyses, evaluated publication bias, and graded evidence quality.

**Results:**

Nine studies (998 patients) were included (four RCTs, five cohort studies). HPT was associated with longer phototherapy duration (SMD = 0.55, 95% CI: 0.06–1.04, *P* = 0.03). Cohort study subgroup analysis yielded consistent results (SMD = 0.90; 95% CI: 0.69 to 1.11, *P* < 0.001, *I*^*2*^ = 39%); the RCTs were not significantly different (SMD = -0.04; 95% CI: -0.15 to 0.08, *P* = 0.54, *I*^*2*^ = 0%). Hospital readmission was higher with HPT (RR = 4.61; 95% CI: 1.43–14.86, *P* = 0.01). Daily bilirubin reduction (WMD = -0.12, 95% CI: -0.68 to 0.44, *P* = 0.68) or complications were not significantly different (RR = 2.29; 95% CI: 0.31–16.60, *P* = 0.41). The evidence quality was very low. HPT was associated with lower parental stress (SMD = -0.44, 95% CI: -0.71 to -0.16, *P* = 0.002). None of three included studies reported exchange transfusion.

**Conclusions:**

The current evidence does not strongly support HPT efficacy for neonatal hyperbilirubinemia, as high-quality data on long-term outcomes are scarce. Future research should prioritize well-designed, large-scale, high-quality RCTs to comprehensively assess HPT risks and benefits.

**Supplementary Information:**

The online version contains supplementary material available at 10.1186/s13052-024-01613-0.

## Background

Of the 140 million newborns born globally each year, approximately 84–112 million will develop jaundice within the first 2 weeks of life[1, 2]. Jaundice affects at least 60% of preterm infants and 80% of infants. Some newborns may develop bilirubin encephalopathy and kernicterus due to high bilirubin levels, which lead to long-term consequences such as cerebral palsy and intellectual developmental disorders. Such consequences impose a significant burden on both society and families [3]. Phototherapy has been the treatment of choice for neonatal hyperbilirubinemia since the late 1940s [4]. Phototherapy alters the molecular structure of bilirubin and produces water-soluble isomers that can be excreted through urine. It is a therapeutic method that can effectively reduce bilirubin levels in neonates [5]. Phototherapy is primarily used in hospitals as part of traditional treatment protocols. However, this can lead to the separation of neonates from their parents, resulting in breastfeeding challenges and heightened parental stress. Simultaneously, the cost of neonatal hospitalization is high, which imposes another significant burden on parents and society. To improve these problems, the United States began to research home phototherapy (HPT) as early as the late 1970s [6].

Most national guidelines remain cautious due to insufficient evidence of safety regarding HPT. For example, the UK national guideline is silent regarding HPT [1], while the 2004 American Academy of Pediatrics (AAP) hyperbilirubinemia guidelines indicated that because the devices available for HPT may not provide the same degree of irradiance or surface-area exposure as those available in the hospital, HPT should only be considered when the gestational age is ≥ 38 weeks and the total serum bilirubin (TSB) is 2–3 mg/dL below the therapeutic threshold [7]. Although evidence for the safety of HPT is insufficient [8], increased economic pressure, the drive for early maternal discharge, the need for family-centered care (FCC) [9], the renewal of phototherapy equipment [10], and increased parental willingness has resulted in an increasing number of people seeking HPT [11, 12]. Several recently published high-quality articles on HPT provided more evidence of HPT safety for increased healthcare confidence. A clinical trial conducted with HPT cohort in the Seattle-Tacoma-Bellevue in 2020 reported that HPT could be successful treated hyperbilirubinemia in the vast majority of the infants [13]. A randomized clinical trial (RCT) conducted in Sweden determined that HPT cost less than IPT while being similarly beneficial for neonates older than 36 gestational weeks, and a follow-up revealed that HPT enhanced parent–child bonding and decreased parental stress when compared to standard hospital care. This suggested that healthcare professionals should consider offering HPT to unimmunized term newborns with hyperbilirubinemia [14, 15]. According to the largest cohort study of newborn hyperbilirubinemia in the UK, HPT may be administered to a small number of children and be just as successful as inpatient phototherapy (IPT), and potentially prevents occupancy of acute beds, aids in FCC delivery, and is viewed positively by parents [16]. Based on the above research, the 2022 AAP hyperbilirubinemia guidelines was updated that for newborn infants who have already been discharged and then develop a TSB above the phototherapy with a home light-emitting diode (LED) based phototherapy device rather than readmission to the hospital is an option for infants who meet the criteria. Additionally, HPT is not advised for newborns with any risk factors for hyperbilirubinemia neurotoxicity [17].

Currently, there are three systematic reviews of HPT. A systematic evaluation by Malwade and Jardine in 2014 concluded that no high-quality clinical trials had compared HPT with conventional IPT and recommended that RCTs be conducted [8]. Ten thematic evaluations on HPT feasibility were performed in 2016, but yielded no high-quality research to support or contradict HPT [18]. In 2020, a meta-analysis of four articles published before 2015 demonstrated that HPT was more effective for treating neonatal hyperbilirubinemia than IPT [19]. Therefore, HPT feasibility and effectiveness remain controversial.

Given that recently published studies have not been considered in any previous meta-analyses to date and have not summarized the risk outcomes for HPT, the interpretation of existing data might change. Against this background, it is necessary to assess the available evidence and evaluate the existing gaps qualitatively and critically. Therefore, we conducted the present systematic review and meta-analysis to examine the risks and advantages of HPT against IPT for treating newborn hyperbilirubinemia.

## Methods

This meta-analysis and systematic review aimed to evaluate the effectiveness of HPT for treating neonatal hyperbilirubinemia in comparison to IPT. The investigation was conducted in accordance with Preferred Reporting Items for Systematic Reviews and Meta-Analysis (PRISMA) statement guidelines [20]. The study was registered with the International Prospective Register of Systematic Reviews (PROSPERO; registration number: CRD42023446531).

### Data sources and search strategy

We systematically searched PubMed, the Cochrane Library, Embase, Web of Science, Wanfang Database, the Chinese National Knowledge Infrastructure Database (CNKI), and the Chinese Science and Technique Journal Database (VIP) from database inception to June 2, 2023 without language or date restrictions. We also searched the International Clinical Trial Registry Platform (ICTRP) and ClinicalTrials.gov trial registries for both completed and ongoing trials. Finally, we manually examined the bibliographies of identified articles to identify additional eligible trials. A search plan was developed using a combination of medical subject headings (MeSH) and free-text terms related to Hyperbilirubinemia, newborn infants, phototherapy, and Home Care Services. Additional file S1 presents the comprehensive search plan.

### Eligibility criteria

All included studies satisfied the following inclusion criteria: (1) population: newborn infants up to 28 days of age with jaundice who required phototherapy; (2) intervention: phototherapy undertaken in the home setting; (3) comparison: phototherapy undertaken in the hospital setting; (4) primary outcomes: phototherapy duration, daily bilirubin level reduction, exchange transfusion; secondary outcomes: hospital readmission, parental stress scale, complications; and (5) study design: RCT or cohort study. Additional file S2 contains the details of the eligibility criteria and outcome definitions.

The exclusion criteria were as follows: (1) lack of a control group, or the control group was not IPT; (2) incomplete data; (3) study involved the same participants but different purposes; and (4) non-original research (meta-analyses, systematic reviews, reviews, guidelines, editorials, animal experiments, pilot studies, commentaries, case reports).

### Study selection

The obtained articles were exported to EndNote 20 reference management software to eliminate duplicated studies. Two researchers conducted a literature review based on the screening criteria. First, the article title and abstract were examined to exclude articles that did not fulfill the requirements, then the full text was read. Articles that contained insufficient information and participants for whom comprehensive data could not be obtained were excluded from the study. Any disagreements between the two investigators were resolved by discussion with a third investigator.

### Data extraction

Data were extracted with a standardized Microsoft Excel data extraction form. Both review authors extracted the data independently and resolved differences by discussion when required. Once missing data had been identified, the original study investigators were contacted to request additional information or data if required. The two researchers extracted the following data from the included studies: (1) study details (publication date, author names, study design, study period, setting, recruitment, funding, country); (2) patient characteristics (sample size, birth weight, gestational age, serum bilirubin at inclusion, emitting materials, maximum irradiance); (3) outcome measures and analyses; (4) study inclusion criteria and guidelines.

### Risk of bias and grade certainty assessment

Two assessors evaluated the potential for bias separately, considering both the articles and protocols. Any discrepancies were discussed and a third reviewer was engaged to assist, if required. Randomized trials were assessed with the Cochrane Collaboration Risk of Bias tool (RoB). The RoB evaluation encompasses six areas: randomization method, allocation scheme, blinding, reporting of loss to follow-up, selection bias, and other biases [21]. The risk of bias in the observational cohort studies was assessed using the Newcastle–Ottawa scale (NOS), which features three grouping items: selection, comparability, and outcomes [22]. Additional file S3 presents the guidelines for evaluating the grading method quality. The evidence quality of the included studies was evaluated using the Grading of Recommendations Assessment, Development, and Evaluation method (GRADEpro Guideline Development Tool, gradepro.org) [23].

### Statistical analysis

More detailed statistical analyses with raw data were conducted using Stata 15 (Stata Corp.) and Review Manager 5.4 (The Cochrane Library). The results were assessed using forest plots. The risk ratio (RR) served as the effect measure for categorical data, while the standardized mean difference (SMD) or weighted mean difference (WMD) served as the effect measure for continuous data. A significance level of *P* < 0.05 was deemed statistically significant. A point estimate and a 95% confidence interval (CI) were used for every effect size. The inconsistency index (*I*^*2*^) test was used for heterogeneity analysis of the included study results. *I*^*2*^ ≤ 50% and *P* ≥ 0.1 indicated no significant heterogeneity among the studies, and the fixed-effects model was used for analysis, while *I*^*2*^ > 50% and *P* < 0.1 indicated significant heterogeneity among the studies, and the random-effects model was used for analysis. To explore the sources of heterogeneity, we conducted subgroup analyses stratified by study type, gestational age at inclusion, serum bilirubin at inclusion, and emitting materials (Additional file S4). To ensure the stability and consistency of our findings, we conducted a sensitivity analysis by excluding one study at a time. Publication bias was evaluated by visual examination of a funnel plot and quantification using Egger’s test. Statistical significance was determined by setting a threshold of < 0.05 for the two-sided *P*-value.

## Results

### Literature search and study selection

**Fig. 1:**
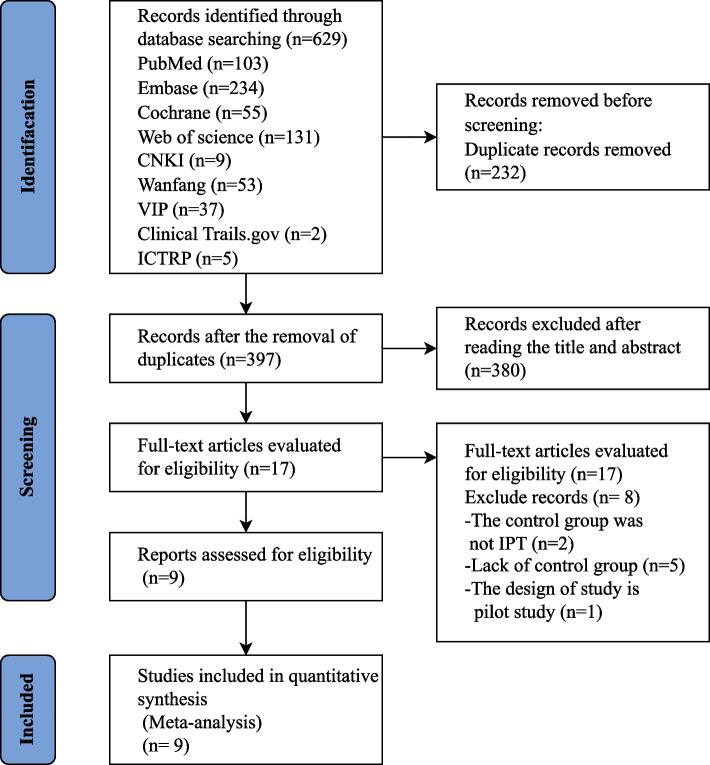
PRISMA flow diagram

A total of 629 publications were identified, of which 232 were excluded due to duplication. We screened 397 titles and abstracts and excluded 380 based on the inclusion and exclusion criteria, leaving 17 full-text articles to be assessed for eligibility. Following the full-text screening, eight articles were eliminated as they did not fulfill the inclusion criteria (Additional file S5) contains the explanations for their exclusion). A total of nine studies [14, 16, 24–30] met the inclusion criteria for the comprehensive review and meta-analysis (Fig. 1).

### Characteristics of the included studies

The nine included studies [14, 16, 24–30] involved 998 participants (579 HPT and 419 IPT). The mean serum bilirubin at inclusion was 16.3 mg/dL. The nine studies involved 58–323 samples and were conducted in six different countries. Four studies (44.4%) were RCTs [14, 27–29] and five (55.6%) were cohort studies[16, 24–26, 30]. The studies assessed HPT efficacy via primary or secondary outcomes. The heterogeneity might have been due to the study type, gestational age at inclusion, serum bilirubin at inclusion, and emitting materials. The HPT inclusion criteria and guidelines were established at the individual study investigators’ discretion (Additional file S6). Table 1 describe the listed research in detail.

**Table 1:** Characteristics of the included studies

Author	Year	Study period	Recruitment	Funding	Country	HPT group/IPT control group							Outcomes	Quality status
						**Sample size**	**Birth weight** **(g**^**†**^**)**	**Gestational age** **(weeks**^**†**^**)**	**Age at inclusion** **(days**^**†**^**)**	**Serum bilirubin at inclusion (mg/dL**^**†**^**)**	**Emitting material**	**Maximum irradiance** **(μW/cm**^**2**^**/nm**^**†**^**)**		
**RCTs**														
Pettersson, M	2021	2016.08–2019.09	Prospective	Yes	Sweden	78/69	3598/3586	39/39	4.10/4.10	20.9/21.1	LED/NA	35/30	①③④	L
	2022												⑤	M
Sardari, S	2019	2017.01–2017.06	Prospective	Yes	Iran	32/32	NA	NA	NA	16.0/16.6	NA	NA	①④⑥	H
Namnabati, M													⑤	H
**Cohort**														
Khajehei, M	2022	2019.01–2019.12	Retrospective	No	Australia	243/80	3228/3347	38^+6^/38^+4^	3.29/3.75	17.0/18.1	LED/LED	35/35	①③	M
Coquery, S. S	2022	2018.01–2020.01	Retrospective	No	France	39/100	3185/3287	38^+6^/39	4.58/5.00	NA	LED/NA	35/NA	④	L
Noureldein, M	2021	2018.04–2020.09	Retrospective	No	UK	100/50	3008/2897	37/37	5.00/5.00	15.9/16.0	LED/NA	35/NA	①②④	M
Eggert, L. D	1985	1981.08–1982.01	Prospective	No	USA	62/55	NA	NA	4.20/3.00	15.3/12.0	Fluorescent/ Fluorescent	NA	①②④⑥	M
Slater, L	1984	1979.10–1981.08	Prospective	Yes	USA	25/33	NA	39^+5^/39^+4^	3.92/3.35	16.5/15.8	Fluorescent/NA	NA	①③④⑥	L

^†^Median (interquartile range [IQR]) or mean (SD)

① Phototherapy duration; ② Daily bilirubin level decrease; ③ Exchange transfusion; ④ Hospital readmission; ⑤ Parental Stress Scale; ⑥ Complications

*HPT* Home phototherapy, *IPT*  inpatient phototherapy, *RCT*  randomized controlled trial, *LED*  light-emitting diode, *L*  low risk of bias, *M* medium risk of bias/some concerns, *H*  high risk of bias, *NA* not applicable

### Assessment of risk of bias

The RoB [21] determined that two trials [27, 29]had a high risk of bias, where each study had between one and two of the seven possible sources of bias. Participant and staff blinding was most often subject to bias, followed by outcome evaluation blinding and random sequence creation. One study demonstrated the lowest possible chance of bias in every category [28]. The cohort studies were of low to medium quality. Additional files S7 and S8 present the quality assessment findings for each study.

### Primary outcomes

#### Phototherapy duration

**Fig. 2:**
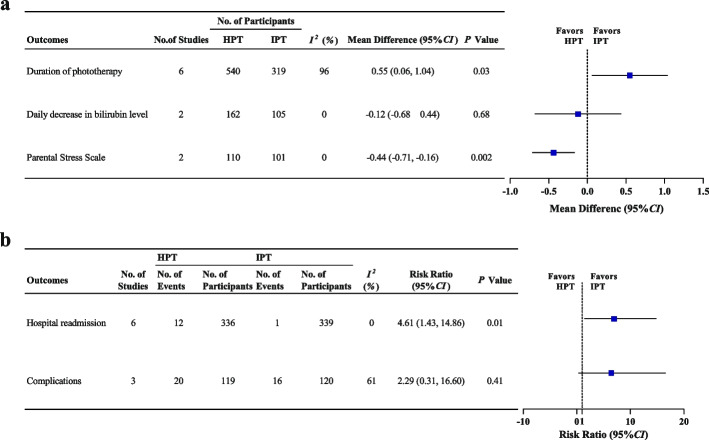
Summary of primary and secondary outcomes meta-analysis of HPT vs. IPT

Six studies [16, 25, 26, 28–30] (859 neonates) compared the phototherapy duration between HPT and IPT. The *I*^*2*^ test statistics for these studies revealed a substantial degree of heterogeneity (*I*^*2*^ = 96%, *P* < 0.001). Therefore, the data were analyzed with the random-effects model. The meta-analysis demonstrated that HPT duration was significantly longer (SMD = 0.55, 95% CI = 0.06–1.04, *P* = 0.03) than that of IPT (Fig. 2a).

#### Daily bilirubin level reduction

Two studies [16, 25] (267 neonates) compared daily bilirubin level reductions after HPT or IPT treatment. There was no significant heterogeneity between the groups (*I*^*2*^ = 0%, *P* = 0.94). Therefore, the data were examined using the fixed-effects model. The meta-analysis revealed no difference between HPT and IPT in terms of daily bilirubin level reduction (WMD = -0.12, 95% CI = -0.68 to 0.44, *P* = 0.68). Figure 2a depicts the forest plot of the meta-analysis.

#### Exchange transfusion

Three studies [26, 28, 30] (528 neonates) compared the incidence of exchange transfusion after HPT or IPT. No exchange transfusions were conducted on newborns following either HPT or IPT.

### Secondary outcomes

#### Hospital readmission

Six studies [16, 24, 25, 28–30] (675 neonates) compared the incidence of hospital readmission after HPT or IPT. There was no significant heterogeneity between the groups (*I*^*2*^ = 0%, *P* = 0.83). The hospital readmission rate in the HPT group and IPT group was 3.57% (12/336) and 0.29% (1/339), respectively. The IPT group had a considerably lower hospital readmission incidence rate than the HPT group (RR = 4.61; 95% CI: 1.43–14.86, *P* = 0.01) (Fig. 2b**)**.

#### Parental stress scale

Two studies [14, 27] (211 neonates) compared parental stress scales after HPT or IPT. There was no significant heterogeneity between the groups (*I*^*2*^ = 0%, *P* = 0.45). The data were analyzed using the random-effects model considering the various measurement scales. The parents of neonates who received HPT had significantly lower stress than those of parents of neonates who received IPT (SMD = -0.44, 95% CI = -0.71 to -0.16, *P* = 0.002) (Fig. 2a).

#### Complications

Three studies [25, 29, 30] (239 neonates) compared the incidence of complications between HPT and IPT. There was no discernible difference between the two groups in the incidence of complications [HPT: 16.81% (20/119), IPT: 13.33% (16/120, RR = 2.29; 95% CI: 0.31–16.60, *P* = 0.41) (Fig. 2b).

### Certainty of evidence

The level of certainty regarding the outcomes was assessed with the GRADE method [23]. The analysis indicated that the level of evidence supporting parental stress alone was moderate, while the general level of evidentiary quality was deemed very low primarily due to the high risk of bias, small sample sizes, and wide CI. A summary of findings in Additional file S9 presents the results and evaluations.

### Sensitivity analysis

Sensitivity analyses were performed for the outcomes (phototherapy duration, hospital readmission) of HPT vs. IPT. The sensitivity analysis revealed a small difference between the combined effect value and the total combined effect value, indicating that our results were stable (Additional file S10).

### Subgroup analysis

**Fig. 3:**
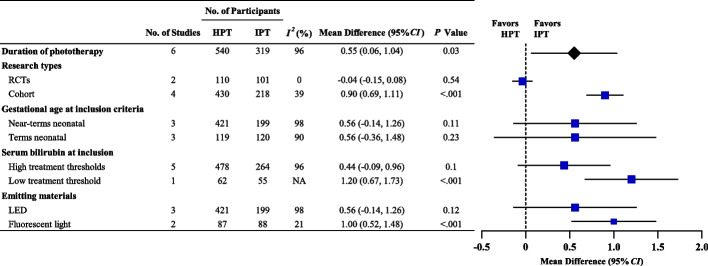
Subgroup analysis of phototherapy duration for HPT vs. IPT

The phototherapy duration among the included studies was very heterogeneous (*I*^*2*^ = 96%, *P* < 0.001). Accordingly, subgroup analysis was conducted according to the study type, gestational age at inclusion, serum bilirubin at inclusion, and emitting materials to investigate the potential sources of heterogeneity. For the study type subgroup, two RCTs [28, 29] (211 neonates) and four cohort studies [16, 25, 26, 30] (648 neonates) reported the outcome of phototherapy duration. In both RCTs, the phototherapy duration did not vary significantly (SMD = -0.04; 95% CI: -0.15 to 0.08, *P* = 0.54, *I*^*2*^ = 0%). The four cohort studies demonstrated that HPT duration was statistically significantly longer than that of IPT (SMD = 0.90; 95% CI: 0.69–1.11, *P* < 0.001,* I*^*2*^ = 39%). The subgroup analyses according to gestational age at inclusion, serum bilirubin at inclusion, and emitting materials did not demonstrate any evidence of effect modification (Fig. 3).

### Publication bias

Publication bias was assessed with a funnel plot and Egger’s test. Subjective indication of publication bias was observed through asymmetrical distribution in the funnel plot (Additional file S11). Nevertheless, Egger’s test indicated the absence of significant publication bias (*P* = 0.298). The funnel plot and Egger’s test indicated no significant evidence of publication bias for hospital readmission (*P* = 0.127).

## Discussion

This systematic review and meta-analysis included nine studies (RCTs and cohort studies) that involved 998 participants. Overall, the primary outcome demonstrated that HPT duration was longer than that of IPT, but there was no significant difference in the daily bilirubin level reduction, and neither group had exchange transfusion cases. The secondary outcomes demonstrated that HPT was followed by higher hospital readmission rates than IPT, but parental stress was lower, and there was no significant difference in phototherapy complications.

### Primary outcomes

#### Phototherapy duration

An earlier systematic review reported no significant difference in the phototherapy duration between HPT and IPT [19]. Contrastingly, our findings were supported by very low-quality evidence that HPT duration is longer than that of IPT. The subgroup analysis revealed similar findings, with the cohort studies demonstrating heterogeneity. A UK cohort study suggested this heterogeneity could be explained by greater parental adherence to treatment due to direct patient supervision and more frequent serum bilirubin checks at the hospital [16]. Cost and separation are also factors that affect the phototherapy duration in hospitals [30]. Conversely, infants in the hospital setting often receive double or triple phototherapy that provides higher light irradiance and faster TSB reduction [31]. However, the combined RCT subgroup findings did not reveal a meaningful difference between the two groups. This might have been related to the rigorous definition and duration of phototherapy interruption time in prospective RCTs [28]. Therefore, it is important to interpret these findings with caution as most of the observational studies were conducted retrospectively, which might have introduced selection and information bias. Nevertheless, the RCTs demonstrated higher methodological quality and certainty of evidence than the cohort studies. Therefore, more large, well-conducted RCTs would resolve this question.

#### Daily bilirubin level reduction

The TSB is used as the gold standard test to inform choices about phototherapy and level of care escalation [17]. Our findings were consistent with the results of a meta-analysis and cohort study that compared the daily bilirubin level reduction following HPT and IPT. Contrastingly, Zainab and Adlina reported that HPT had a higher reduction rate than IPT [32]. The intensity of phototherapy supplied, such as the spectrum of light emitted, spectral irradiance delivered to the skin, and spectral power, determines phototherapy success [7, 17]. In this study, subgroup analysis could not be applied to the included studies; therefore, future studies should explore the optimal use of HPT devices from different aspects.

#### Exchange transfusion

Immediate exchange transfusion is warranted when phototherapy has failed to effectively reduce the rate of bilirubin rise and the TSB or transcutaneous bilirubin measurement nears or exceeds exchange concentrations, or if the infant demonstrates any signs of moderate to advanced acute bilirubin encephalopathy [1, 17, 33]. As there were no cases of exchange transfusion in three of the included studies [26, 28, 30], meta-analysis was not possible. Although the inclusion criteria varied for each study (e.g., gestational age, chronological age, TSB, guidelines followed), no cases of exchange transfusion occurred, where the possible reasons were: (1) most of the studies excluded individuals with risk characteristics and only included a limited number of participants; (2) daily bilirubin monitoring and 24/7 medical support; and (3) high family compliance.

### Secondary outcomes

#### Hospital readmission

Our meta-analysis determined that HPT was followed by a 4.61-fold higher rate of hospital readmissions than IPT. The HPT readmission rate in six included studies was 0 ~ 8%, while that for IPT was 0 ~ 2% [16, 24, 25, 28–30]. Parents’ worries about treatment failure or non-compliance led to HPT readmissions. The other causes for HPT readmission were poor hydration or unrelated to hyperbilirubinemia [30]. One RCT had a 4% readmission rate, which was probably due to the strict information protocol that included daily hospital visits and 24/7 telephone support [28]. IPT might reduce readmission by prolonging hospital stays. Furthermore, differences in the definition of readmissions might have affected this outcome. Accordingly, future studies should be strictly defined as the rate of rehospitalization within 7 days of discharge for phototherapy recommencement.

#### Parental stress scale

More than 25% of parents of children hospitalized in pediatric (non-intensive care) wards experienced significant post-traumatic stress symptoms after their child was discharged [34]. HPT reduces parental stress by reducing disruption to mother–infant bonding and breastfeeding programs and avoiding separation from the infant [35]. Consistent with our findings, the parents of infants who received HPT reported lower stress levels compared with the parents of infants who received IPT. However, two of the included RCTs used different parental stress scales and measurement times. The scale of Namnabati et al. was derived from the Haidari, Hassanpour, and Fouladifrom scales [27, 36]. After the intervention, the mothers completed the questionnaires. Pettersson et al*.* based their study on the Swedish Parenthood Stress Questionnaire, in which parents completed the questionnaire 4 months after the intervention ended [14, 37–39]. More research is needed to explore the short- and long-term social and psychological development effects of HPT on parents and infants.

#### Complications

Currently, some medical staff are concerned that parents’ lack of medical knowledge might result in complications from inadequate nursing care, such as corneal abrasion, eye patch misuse, excessive weight loss, dehydration, diarrhea, or temperature derangements, and therefore prefer IPT. However, our findings suggested that HPT complications are similar to that of IPT. The study criteria were designed to select infants at low risk of developing complicating illnesses and to select families capable of providing the appropriate nursing care [30]. Comprehensive instructions for use and guidelines for identifying the adverse effects of phototherapy are available. Furthermore, parents are required to regularly monitor the relevant data and record it in the flow sheet [25], and 24/7 medical support is available [28]. Additionally, social factors might also be one of the reasons parents are currently more willing to care for their infant at home and demonstrate better compliance. The increase in information in the Internet age has increased public and family awareness, which renders parents more sensitive to the treatment of newborns. Simultaneously, the global fertility rate has led to a decline in the number of children per family, and parents focus more attention on their children [40].

### Strengths and limitations

The strengths off this meta-analysis are as follows: (1) compared to earlier findings, our cumulative sample size was the greatest to date; (2) cohort studies and RCTs were included; (3) primary and secondary outcomes were analyzed and sources of heterogeneity were explored via subgroup analyses; and (4) the certainty of evidence was scored using the GRADE method.

The limitations of our meta-analysis are as follows: (1) most of the included articles were retrospective, which increased the risk of information and selection bias; furthermore, the included RCTs featured relatively small sample sizes; (2) the clinical heterogeneity of the studies, especially the large variation in participants’ personal traits, HPT protocols, and equipment used, might have led to serious heterogeneity; (3) evidence for the long-term outcomes and cost-effectiveness of interventions was lacking; and (4) the very low certainty of the evidence for all outcomes.

## Conclusions

Overall, given that an RCT has a higher level of evidence than a cohort study, there was no evidence to support the premise that HPT duration is longer than that of IPT. HPT might not be inferior to IPT in terms of daily bilirubin level reduction and complications. Parental stress following HPT was relatively lower, but the readmission rate might be higher. The current evidence does not strongly support the efficacy of HPT for neonatal hyperbilirubinemia, where there is a paucity of high-quality data on long-term outcomes. Future research should prioritize well-designed, large-scale, high-quality RCTs to comprehensively assess the risks and benefits of HPT.

### Supplementary Information

**Additional file 1:** **Additional file S1.** Search strategies. **Additional file S2.** Eligibility criteria during selection. **Additional file S3.** Criteria for grading methodological quality. **Additional file S4.** Basis for grouping in sub-analysis. **Additional file S5.** List of references with final exclusion reasons. **Additional file S6.** Study inclusion criteria and guidelines. **Additional file S7.** Cochrane RoB to rate the risk of bias in RCTs. **Additional file S8.** The NOS to rate the risk of bias in cohort and case-control studies. **Additional file S9.** Evidence profiles. **Additional file S10.** Sensitivity analysis. **Additional file S11. **Funnel plot.

## Data Availability

The datasets used and/or analyzed during the current study are available from the corresponding author upon reasonable request.

## Abbreviations

HPT: Home phototherapy
AAP: The American Academy of Pediatrics
TSB: Total serum bilirubin
FCC: Family-centered care
RCTs: Randomized clinical trials
IPT: Inpatient phototherapy
LED: Light-emitting diode
RR: Risk Ratio
SMD: Standardized mean difference
WMD: Weighted mean difference
CI: Confidence interval
I^2^: Inconsistency index
