# Supplementary material for: Efficacy of home phototherapy versus inpatient phototherapy for neonatal hyperbilirubinemia: a systematic review and meta-analysis
Source: Ital J Pediatr. 2024 Mar 4;50:37. doi: 10.1186/s13052-024-01613-0 (PMC10910825; doi:10.1186/s13052-024-01613-0)

**Efficacy of home phototherapy versus inpatient phototherapy for** **neonatal hyperbilirubinemia: a systematic review and meta-analysis**

**Contents of** **Additional File:**

**Additional File S1.** **Search strategies.**

**Additional File S2.** **Eligibility criteria during selection**

**Additional File S3. Criteria for grading methodological quality**

**Additional File S4. Basis for grouping in sub-analysis**

**Additional File S5.** **List of references with final exclusion reasons**

**Additional File S6. Study inclusion criteria and guidelines**

**Additional File S7. Cochrane RoB to rate the risk of bias in RCTs**

**Additional File S8. The NOS to rate the risk of bias in cohort and case-control studies**

**Additional File S9. Evidence profiles**

**Additional File S10. Sensitivity analysis**

**Additional File S11.** **Funnel plot**

**Additional File S1. Search strategies**

**PubMed**

**Search Query**

**#1** ((((("Hyperbilirubinemia"[Mesh] OR "Hyperbilirubinemia, Neonatal"[Mesh] OR "Jaundice"[Mesh] OR "Jaundice, Obstructive"[Mesh] OR "Jaundice, Neonatal"[Mesh] OR "Kernicterus"[Mesh]) OR (hyperbilirubinemi*[TW] OR hyperbilirubinaemi*[TW] OR bilirubinemi*[TW] OR bilirubinaemi*[TW] OR jaundice[TW] OR jaundices[TW] OR jaundiced[TW] OR kernicterus[TW] OR icter*[TW] OR (encephalopath*[TW] AND bilirubin[TW]))))))

**#2** ((((infant, newborn[MeSH] OR newborn*[TIAB] OR "new born"[TIAB] OR "new borns"[TIAB] OR "newly born"[TIAB] OR baby*[TIAB] OR babies[TIAB] OR premature[TIAB] OR prematurity[TIAB] OR preterm[TIAB] OR "preterm"[TIAB] OR "low birth weight"[TIAB] OR "low birthweight"[TIAB] OR VLBW[TIAB] OR LBW[TIAB] OR infant[TIAB] OR infants[TIAB] OR infantile[TIAB] OR infancy[TIAB] OR neonat*[TIAB]))))

**#3** ((((((((((((((((((Phototherapy[Title/Abstract]) OR (biliblanket[Title/Abstract])) OR (wallaby[Title/Abstract])) OR (fiber optic[Title/Abstract])) OR ("Phototherapy"[Mesh])) OR (((("Heliotherapy"[Mesh] OR "Sunlight"[Mesh] OR heliotherapy[TW] OR sunlight[TW] OR "sun light"[TW] OR sunshine[TW] OR "sun shine"[TW] OR sun*[TW] OR solar*[TW] OR window*[TW] OR daylight[TW] OR "day light"[TW]))))) OR (Phototherapies[Title/Abstract])) OR (Therapy, Photoradiation[Title/Abstract])) OR (Photoradiation Therapies[Title/Abstract])) OR (Therapies, Photoradiation[Title/Abstract])) OR (Light Therapy[Title/Abstract])) OR (Light Therapies[Title/Abstract])) OR (Therapies, Light[Title/Abstract])) OR (Therapy, Light[Title/Abstract])) OR (Photoradiation Therapy[Title/Abstract])))))

**#4** ((((home[Title/Abstract]) OR (house[Title/Abstract])) OR (Home Care Agencies[Title/Abstract])) OR (((("Home Care Services"[Mesh]) OR ("Home Care Services, Hospital-Based"[Mesh])) OR ((((((((((Home Care Services[Title/Abstract]) OR (Home Care Service[Title/Abstract])) OR (Service, Home Care[Title/Abstract])) OR (Services, Home Care[Title/Abstract])) OR (Care Services, Home[Title/Abstract])) OR (Domiciliary Care[Title/Abstract])) OR (Care, Domiciliary[Title/Abstract])) OR (Home Health Care[Title/Abstract])) OR (Home Care[Title/Abstract])) OR (Care, Home[Title/Abstract]))) OR (((((((((((((((((Home Care Services, Hospital-Based[Title/Abstract]) OR (Cares, Hospital-Based Home[Title/Abstract])) OR (Cares, Hospital Based Home[Title/Abstract])) OR (Home Care, Hospital-Based[Title/Abstract])) OR (Home Care, Hospital Based[Title/Abstract])) OR (Home Cares, Hospital-Based[Title/Abstract])) OR (Home Cares, Hospital Based[Title/Abstract])) OR (Hospital Home Care Services[Title/Abstract])) OR (Hospital-Based Home Care[Title/Abstract])) OR (Hospital Based Home Care[Title/Abstract])) OR (Home Care Services, Hospital Based[Title/Abstract])) OR (Hospital-Based Home Care Services[Title/Abstract])) OR (Hospital Based Home Care Services[Title/Abstract])) OR (Care, Hospital-Based Home[Title/Abstract])) OR (Care, Hospital Based Home[Title/Abstract])) OR (Hospital-Based Home Cares[Title/Abstract])) OR (Hospital Based Home Cares[Title/Abstract]))))

**#5 #**1 AND #2 AND #3 AND #4

**Embase**

**No. Query**

**#1** hyperbilirubinemia:ti,ab,kw OR 'neonatal hyperbilirubinemia':ti,ab,kw OR jaundice:ti,ab,kw OR 'obstructive jaundice':ti,ab,kw OR 'newborn jaundice':ti,ab,kw OR kernicterus:ti,ab,kw

**#2** 'hyperbilirubinemia'/exp OR 'hyperbilirubinemia'

**#3** 'jaundice'/exp

**#4** newborn:ti,ab,kw OR baby:ti,ab,kw OR prematurity:ti,ab,kw OR 'low birth weight':ti,ab,kw OR infant:ti,ab,kw

**#5** 'newborn'/exp OR 'newborn'

**#6** phototherapy:ti,ab,kw OR 'bilirubin light':ti,ab,kw OR wallaby:ti,ab,kw OR 'fiber optics':ti,ab,kw OR heliotherapy:ti,ab,kw OR sunshine:ti,ab,kw OR sunlight:ti,ab,kw

**#7** 'phototherapy'/exp OR 'phototherapy'

**#8** 'heliotherapy'/exp OR 'heliotherapy'

**#9** 'sunlight'/exp OR 'sunlight'

**#10** 'home care'/exp OR 'home care'

**#11** 'home'/exp OR 'home'

**#12** home:ti,ab,kw OR house:ti,ab,kw OR 'home care':ti,ab,kw

**#13** #1 OR #2 OR #3

**#14** #4 OR #5

**#15** #6 OR #7 OR #8 OR #9

**#16** #10 OR #11 OR #12

**#17** #13 AND #14 AND #15 AND #16

**Cochrane Library**

**ID Search**

**#1** newborn OR neonat* OR baby OR infant OR premature OR preterm OR low birth weight

**#2** Hyperbilirubinemia OR Jaundice OR Kernicterus

**#3** home OR house OR home care

**#4** phototherapy OR biliblanked OR sunlight OR wallaby OR fiber optic OR heliotherapy

**#5** #1 AND #2 AND #3 AND #4

**Web of Science**

**ID Search**

**#1** TS= (newborn OR neonat* OR baby OR infant OR premature OR preterm OR low birth weight)

**#2** TS= (Hyperbilirubinemia OR Jaundice OR Kernicterus)

**#3** TS= (home OR house OR home care)

**#4** TS= (phototherapy OR biliblanked OR sunlight OR wallaby OR fiber optic OR heliotherapy)

**#5** #1 AND #2 AND #3 AND #4

**China National Knowledge Infrastructure (CNKI) - field searching in Chinese**

**序号 检索表达式**

**#1** 主题：新生儿（精确)

**#2** 主题：家庭（精确）

**#3** 主题：光疗（精确）

**#4** #1 AND #2 AND #3

**Wanfang Datebase - field searching in Chinese**

**序号 检索表达式**

**#1** 主题：(新生儿 or 足月儿）

**#2** 主题：(家庭 or 居家)

**#3** 主题：(光疗 or 蓝光)

**#4** #1 AND #2 AND #3

**China Science and Technology Journal Database (VIP) - field searching in Chinese**

**序号 检索表达式**

**#1** 主题：(新生儿 or 足月儿）

**#2** 主题：(家庭 or 居家)

**#3** 主题：(光疗 or 蓝光)

**#4** #1 AND #2 AND #3

**Clinical Trials.gov**

**Condition or disease Search**

newborn

**Intervention/Treatment Search**

Home phototherapy

**The International Clinical Trials Registry Platform (ICTRP)**

**Search Portal**

neonatal home phototherapy

**Additional File S2. Eligibility criteria during selection**

**Additional File S2 -A Eligibility Criteria**

| **1.** | **Condition treated** | Neonatal Hyperbilirubinemia |
| --- | --- | --- |
| **2.** | **Study population participants** | Newborn (birth ≤28 days) |
| **3.** | **Intervention evaluated** | Home phototherapy (HPT) |
| **4.** | **Comparison** | Inpatient phototherapy (IPT) |
| **5.** | **Outcomes** | - Duration of phototherapy - Hospital readmission - Daily decrease in bilirubin level - Exchange transfusion - Complications - Parental Stress Scale |
| **6.** | **Study design** | Randomized controlled trials or Cohort |

**Additional File S2 -B Definition of outcomes**

|  | **Outcomes** | **Definitions** |
| --- | --- | --- |
| **1.** | Duration of phototherapy | Duration of time exposed to phototherapy (days). Some infants will have short periods off phototherapy for feeding, etc. |
| **2.** | Hospital readmission | Rehospitalisation of discharge for recommencement of phototherapy. |
| **3.** | Daily decrease in bilirubin level | Rate of change of serum bilirubin (mg/dL/d) from initiation of phototherapy to cessation of phototherapy. |
| **4.** | Exchange transfusion | need for exchange transfusion. |
| **5.** | Complications | Phototherapy complications (e.g., skin rashes, diarrhea, fever, weakness, weak lactation, restlessness, omphalitis, conjunctivitis, vomiting, temperature instability). |
| **6.** | Parental Stress Scale | A higher score indicates more stress. |

**Additional File S3. Criteria for grading methodological quality**

| **Assessment quality of RCTs in the Cochrane Reviewer’ Handbook** | |
| --- | --- |
| Low risk of bias | The study is judged to be at low risk of bias for all domains for this result. |
| Some concerns | The study is judged to raise some concerns in at least one domain for this result, but to be at high risk of bias for any domain. |
| High risk of bias | The study is judged to be at high risk of bias in at least one domain for this result.  Or  The study is judged to have some concerns for multiple domains in a way that substantially lowers confidence in the result. |
| **Assessment quality of observational studies with the Newcastle-Ottawa Scales (NOS)** | |
| Low risk of bias | Up to one item was judged inadequate in a study. |
| Medium risk of bias | Up to three items was judged inadequate in a study. |
| High risk of bias | More than three items were judged inadequate in a study. |
| Very high risk of bias | There was no description of methods. |

**Additional File S4. Basis for grouping in sub-analysis**

| **The groups in sub-analysis** | **Contents in each stratification** |
| --- | --- |
| **Research types** |  |
| RCTs | Randomized Controlled Trials |
| Cohort | Cohort study |
| **Gestational age at inclusion criteria (weeks)** | |
| Near-terms neonatal | The inclusion criteria gestational age ≥35weeks. |
| Terms neonatal | The inclusion criteria gestational age ≥37weeks. |
| **Serum bilirubin at inclusion (mg/dL)** |  |
| High treatment thresholds | The treatment threshold is above 15mg/dL. |
| Low treatment threshold | The treatment threshold is below 15mg/dL. |
| **Emitting materials** |  |
| light-emitting diode (LED) | Home phototherapy uses LED light sources. |
| fluorescent light | Home phototherapy uses fluorescent light sources. |

**Additional File S5. List of references with final exclusion reasons**

| **No.** | **Studies** | **First author** | **Reasons of exclusion** | **Publication year** |
| --- | --- | --- | --- | --- |
| **1.** | Pilot study of home phototherapy for neonatal jaundice monitored in maternity ward during the enforced Italy-wide COVID-19 national lockdown | Zanardo, V. | Lack of control group | 2022 |
| **2.** | Biliblanket Utilization for Outpatient Treatment of Newborn Jaundice | Orringer, K. | Comparison between biliblanked use group and no biliblanked group | 2022 |
| **3.** | Home Phototherapy: Challenges, Faults, and Outcomes | Boskabadi, H. | Lack of control group | 2022 |
| **4.** | Evaluation of Home Phototherapy for Neonatal Hyperbilirubinemia | Chang, P. W. | Lack of control group | 2020 |
| **5.** | Low-cost home-use light-emitting-diode phototherapy as an alternative to conventional methods | Yilmaz, A. | Comparison between the home-type phototherapy unit group and conventional blue-light fluorescent phototherapy group | 2015 |
| **6.** | Effectiveness of home versus hospital phototherapy for term infants with uncomplicated hyperbilirubinemia: a pilot study in Pahang, Malaysia | Zainab, K. | The design of study is pilot study. | 2004 |
| **7.** | Home phototherapy for neonatal jaundice--technology and teamwork meeting consumer and service need | Jackson, C. L. | Lack of control group | 2000 |
| **8.** | 14 Years of experience with home phototherapy | Rogerson, A. G. | Lack of control group | 1986 |

**Additional File S6. Study inclusion criteria and guidelines**

| **No.** | **Author** | **Year** | **Inclusion Criteria** | **Guidelines** |
| --- | --- | --- | --- | --- |
| **1.** | Khajehei, M. | 2022 | - Local-catchment mothers. - Birth gestation ≥35 weeks. - Hospital discharge between 4 and 96 h after birth. - Receiving at least one home visit from the midwifery-at-home service. | - Jaundice management (risk assessment, clinical review, transcutaneous bilirubinometer and serum bilirubin) followed the New South Wales Health Guideline 2016. - Gestational age and newborn age bilirubin treatment threshold nomograms identical to those of the National Institute for Health and Care Excellence Guideline 2010 were used. |
| **2.** | Coquery, S. S. | 2022 | - We included term and late-preterm neonates (gestational age at birth ≥35 weeks) . - The inclusion criteria were neonatal jaundice with unconjugated hyperbilirubinemia of identified or strongly suspected etiology in a neonate aged of ≥48 h or more even if jaundice was associated with one or more risk factors for development of severe hyperbilirubinemia . The discharge of these neonates at risk of severe hyperbilirubinemia would not have been possible so early without this program. These risk factors were (i) a jaundice observed in the first 24 h of life, (ii) a blood group incompatibility or other hemolytic diseases, (iii) a gestational age less than or equal to 38 weeks, (iv) a previous sibling with jaundice, a hematoma or significant bruising, (v) an exclusive breastfeeding with nursing difficulties , and (vi) Asian, African, and Indian origin of the parents. - No infant had other neurotoxicity risk factors or symptoms as neurologic signs, acidosis, hypoxia, unstable temperature, or infection. - Neonate’s residence had to be in the hospital at home’s area of intervention. | - The treatment indication curves are recommended by the SFN and the CNRHP that follow the [American Academy of Pediatrics](https://publications.aap.org/pediatrics) recommendations. |
| **3.** | Pettersson, M. | 2022 | - Term neonates aged >48 h with gestational age >36 weeks. - TSB of 300–400 μmol /L (18–23.4 mg/dl). | - Inclusion criteria were based on Swedish national guidelines. - Patients meeting the [American Academy of Pediatrics](https://publications.aap.org/pediatrics) criteria for in-hospital phototherapy. |
| **4.** | Pettersson, M. | 2021 | - Term neonates aged >48 h with gestational age >36 weeks. - TSB of 300–400 μmol/L (18–23.4 mg/dl). | - The Swedish Parenthood Stress Questionnaire (SPSQ) consists of 34 items rated on a 5-point scale; the mean score is calculated for five subscales (incompetence, role restriction, social isolation, spouse relationship problems and health problems), and a higher score indicates more stress. |
| **5.** | Noureldein, M. | 2021 | - Corrected gestational age (CGA) ≥35 weeks. - 2eight ≥2kg. - ≥48hours of age and serum bilirubin (SBR) ≤50 μmol/L above treatment thresholds. | - The National Institute for Health and Care Excellence (NICE) guidelines. |
| **6.** | Sardari, S. | 2019 | - Mature infants. - Physician's confirmation of phototherapy. - Indirect bilirubin greater than 14 and less than 18 mg/day. - More than 3 days of age. - Over 2500 g weight. - Negative Coombs test. - No increase in direct bilirubin. - The absence of risk factors like lethargy, rejection of breastfeeding, fever, blood type incompatibility of the mother and baby, polycythemia, favism, anemia. - History of severe newborn jaundice in the family. - Informed consents were obtained. | - The American Academy of Pediatrics. |
| **7.** | Namnabati, M. | 2019 | - Mature infants. - Physician's confirmation of phototherapy. - Indirect bilirubin greater than 14 and less than 18 mg/day. - More than 3 days of age. - Over 2500 g weight. - Negative Coombs test. - No increase in direct bilirubin. - The absence of risk factors like lethargy, rejection of breastfeeding, fever, blood type incompatibility of the mother and baby, polycythemia, favism, anemia. - History of severe newborn jaundice in the family. | - Parental Stress Scale consisted of 17 items, adapted by Heidari, Hassanpour and Fouladifrom scales such as PSS NICU and Daily Hassles Scale and DOSS-21 with a Cronbach's alpha of 0.87 on a 7-point Likert scale (from 0 to 6). The total score of the scale was calculated by aggregating the score of each item so that the total score range was between 0 and 120. |
| **8.** | Eggert, L. D. | 1985 | - Infant>24 h postnatal age. - Infant birth wt >2,270 g (5 lbs). - Enrolling physician’s assessment that infant's clinical diagnosis and total bilirubin level allowed adequate margin of error for a trial of home phototherapy. - Enrolling physician's assessment that parents were capable of managing home phototherapy. | - NA |
| **9.** | Slater, L. | 1984 | - Term, appropriate-for-gestational-age infant as defined by greater than 37 weeks of gestational age by obstetrical dating and weight of 2,500 to 4,000 g at birth. - Five-minute Apgar score of 7 or greater. - Normal findings on physical examination. - Actively feeding infant. - Stooling and voiding by 24 hours of age. - Greater than 48 hours of age but less than 7 days of age at the initiation of therapy. - Bilirubin levels at the initiation of therapy within the following values: (a) age 2 days, 10 to 15 mg/100 mL; (b) day 3, 13 to 17 mg/100ml;(c) day 4,14to 18mg/100ml; (d)day 5 through 7,15 to 18mg/100ml. - Laboratory values: (a)direct bilirubin<1.5mg/100ml; (b)no evidence of Rh isoimmunization by direct Coombs' test; (c)hematocrit (central)less than 65%; (d)peripheral smear showing no grossly abnormal forms. - Adequate home and parental environment as evaluated by study staff. | - NA |

**Additional File S7. Cochrane RoB to rate the risk of bias in RCTs**


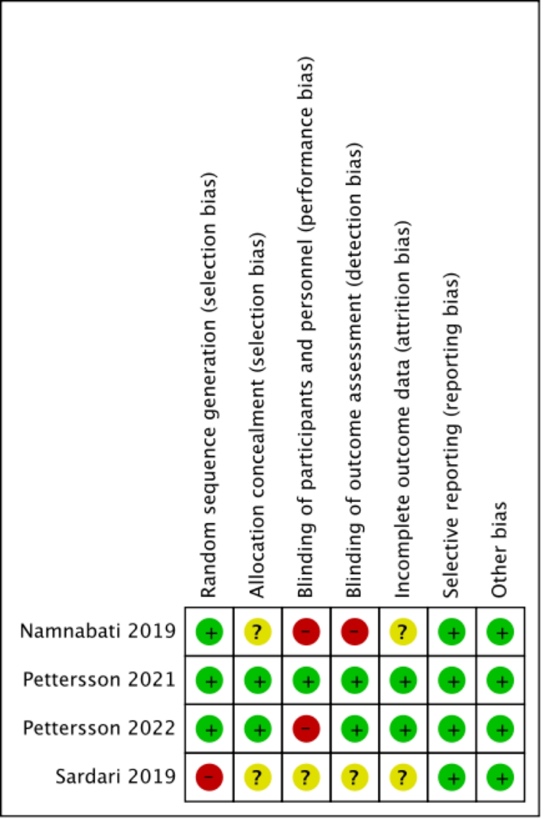

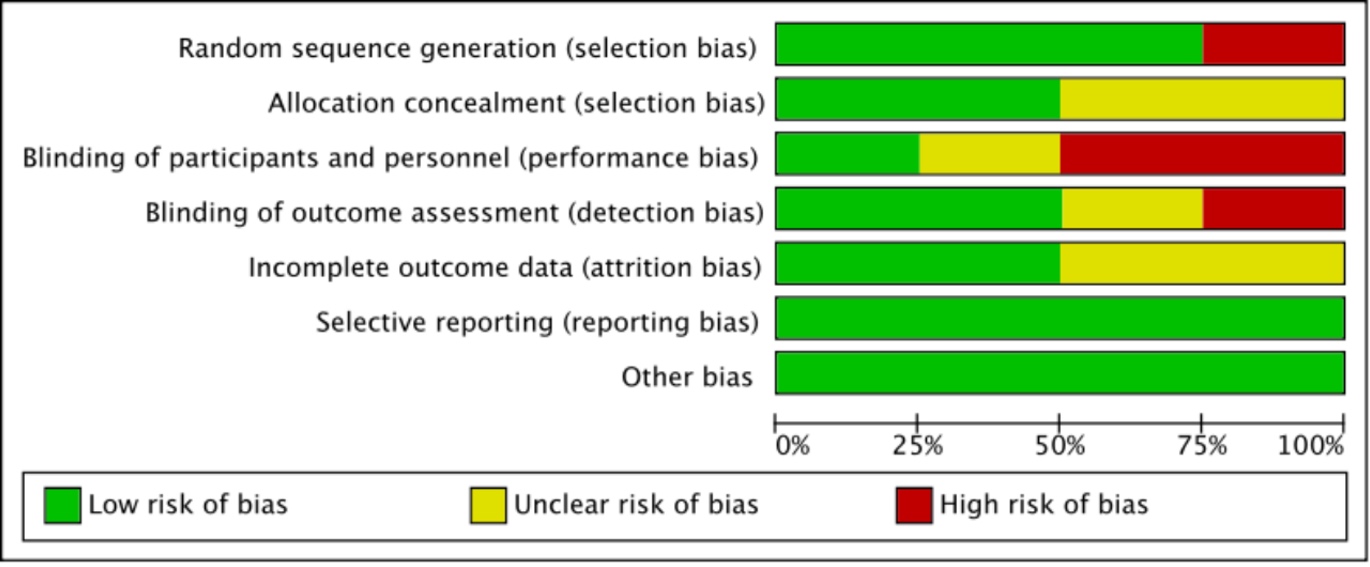


**Additional File S8. The NOS to rate the risk of bias in cohort and case-control studies**

| **References** | **Selection** | | | | **Comparability** | **Outcomes** | | | **Total (max score: 9)** |
| --- | --- | --- | --- | --- | --- | --- | --- | --- | --- |
|  | **Representative-**  **ness of the exposed cohort** | **Selection of the non-exposed cohort** | **Ascertainment of exposure to implants** | **Demonstration**  **that outcome of interest was not present at start of study** | **Comparability**  **of cohorts on the basis of the design or analysis** | **Assessment of outcome** | **Was follow up long enough for outcomes to occur** | **Adequacy of follow up of cohorts** |  |
| Khajehei, M.2022 | **★** | **★** | **★** | **★** | Not described | **★** | **★** | **★** | 7 |
| Coquery, S. S.2022 | **★** | **★** | **★** | **★** | **★★** | **★** | **★** | **★** | 9 |
| Noureldein, M.2021 | **★** | No | **★** | **★** | **★** | **★** | **★** | **★** | 7 |
| Eggert, L. D.1985 | **★** | **★** | **★** | **★** | Not described | **★** | **★** | **★** | 7 |
| Slater, L.1984 | **★** | **★** | **★** | **★** | **★** | **★** | **★** | **★** | 8 |

★ Meet the scoring conditions

**Additional File S9. Evidence profiles**

| **Certainty assessment** | | | | | | | **№ of patients** | | **Effect** | | **Certainty** | **Importance** |
| --- | --- | --- | --- | --- | --- | --- | --- | --- | --- | --- | --- | --- |
| **№ of studies** | **Study design** | **Risk of bias** | **Inconsistency** | **Indirectness** | **Imprecision** | **Other considerations** | **HPT** | **IPT** | **Relative (95% CI)** | **Absolute (95% CI)** |  |  |
| **Duration of phototherapy - RCTs** | | | | | | | | | | | | |
| 2 | randomised trials | serious^a^ | not serious | not serious | very serious^b^ | none | 110 | 101 | - | MD **0.04 lower** (0.15 lower to 0.08 higher) | ⨁◯◯◯ Very low | CRITICAL |
| **Hospital readmission - RCTs** | | | | | | | | | | | | |
| 2 | randomised trials | serious^a^ | not serious | not serious | very serious^b^ | none | 5/110 (4.5%) | 0/101 (0.0%) | **RR 5.62** (0.69 to 46.00) | **0 fewer per 1,000** (from 0 fewer to 0 fewer) | ⨁◯◯◯ Very low | IMPORTANT |
| **Complications - RCTs** | | | | | | | | | | | | |
| 1 | randomised trials | very serious^c^ | serious^f^ | not serious | very serious^b^ | none | 13/32 (40.6%) | 16/32 (50.0%) | **RR 0.81** (0.47 to 1.40) | **95 fewer per 1,000** (from 265 fewer to 200 more) | ⨁◯◯◯ Very low | IMPORTANT |
| **Parental stress scale - RCTs** | | | | | | | | | | | | |
| 1 | randomised trials | serious^a^ | not serious | not serious | serious^e^ | none | 78 | 69 | - | SMD **0.51 lower** (0.84 lower to 0.18 lower) | ⨁⨁◯◯ Low | CRITICAL |

**CI: confidence interval; MD: mean difference; RR: risk ratio; SMD: standardized mean difference**

#### Explanations

**a. lack of blinding**

**b. small trials and wide confidence interval**

**c. due to high risk of bias**

**d. *I^2^*> 75%**

**e. small trials**

**f. Downgraded one level for a single study; unable to assess inconsistency with other studies.**

| **Certainty assessment** | | | | | | | **№ of patients** | | **Effect** | | **Certainty** | **Importance** |
| --- | --- | --- | --- | --- | --- | --- | --- | --- | --- | --- | --- | --- |
| **№ of studies** | **Study design** | **Risk of bias** | **Inconsistency** | **Indirectness** | **Imprecision** | **Other considerations** | **HPT** | **IPT** | **Relative (95% CI)** | **Absolute (95% CI)** |  |  |
| **Duration of phototherapy - Cohort** | | | | | | | | | | | | |
| 4 | observational studies | very serious^c^ | not serious | not serious | not serious | none | 430 | 218 | - | MD **0.9 higher** (0.69 higher to 1.11 higher) | ⨁◯◯◯ Very low | CRITICAL |
| **Hospital readmission - Cohort** | | | | | | | | | | | | |
| 4 | observational studies | very serious^c^ | not serious | not serious | not serious | none | 7/226 (3.1%) | 0/238 (0.0%) | **RR 6.34** (1.19 to 33.69) | **0 fewer per 1,000** (from 0 fewer to 0 fewer) | ⨁◯◯◯ Very low | IMPORTANT |
| **Daily decrease in bilirubin level - Cohort** | | | | | | | | | | | | |
| 2 | observational studies | very serious^a^ | not serious | not serious | very serious | none | 162 | 105 | - | MD **0.12 lower** (0.68 lower to 0.44 higher) | ⨁◯◯◯ Very low | CRITICAL |
| **Complications - Cohort** | | | | | | | | | | | | |
| 2 | observational studies | very serious^c^ | serious^f^ | not serious | very serious^b^ | none | 6/87 (6.9%) | 0/121 (0.0%) | **RR 11.56** (0.67 to 200.54) | **0 fewer per 1,000** (from 0 fewer to 0 fewer) | ⨁◯◯◯ Very low | IMPORTANT |
| **Parental stress scale - Cohort** | | | | | | | | | | | | |
| 1 | observational studies | very serious^c^ | not serious | not serious | very serious^b^ | none | 32 | 32 | - | SMD **0.28 lower** (0.77 lower to 0.21 higher) | ⨁◯◯◯ Very low | CRITICAL |

**CI: confidence interval; MD: mean difference; RR: risk ratio; SMD: standardized mean difference**

#### Explanations

**a. lack of blinding**

**b. small trials and wide confidence interval**

**c. due to high risk of bias**

**d. *I^2^*> 75%**

**e. small trials**

**f. Downgraded one level for moderate inconsistency (heterogeneity).**

**Additional File S10. Sensitivity analysis**

**Additional File S10-A Sensitivity analysis of the duration of phototherapy for HPT VS IPT**


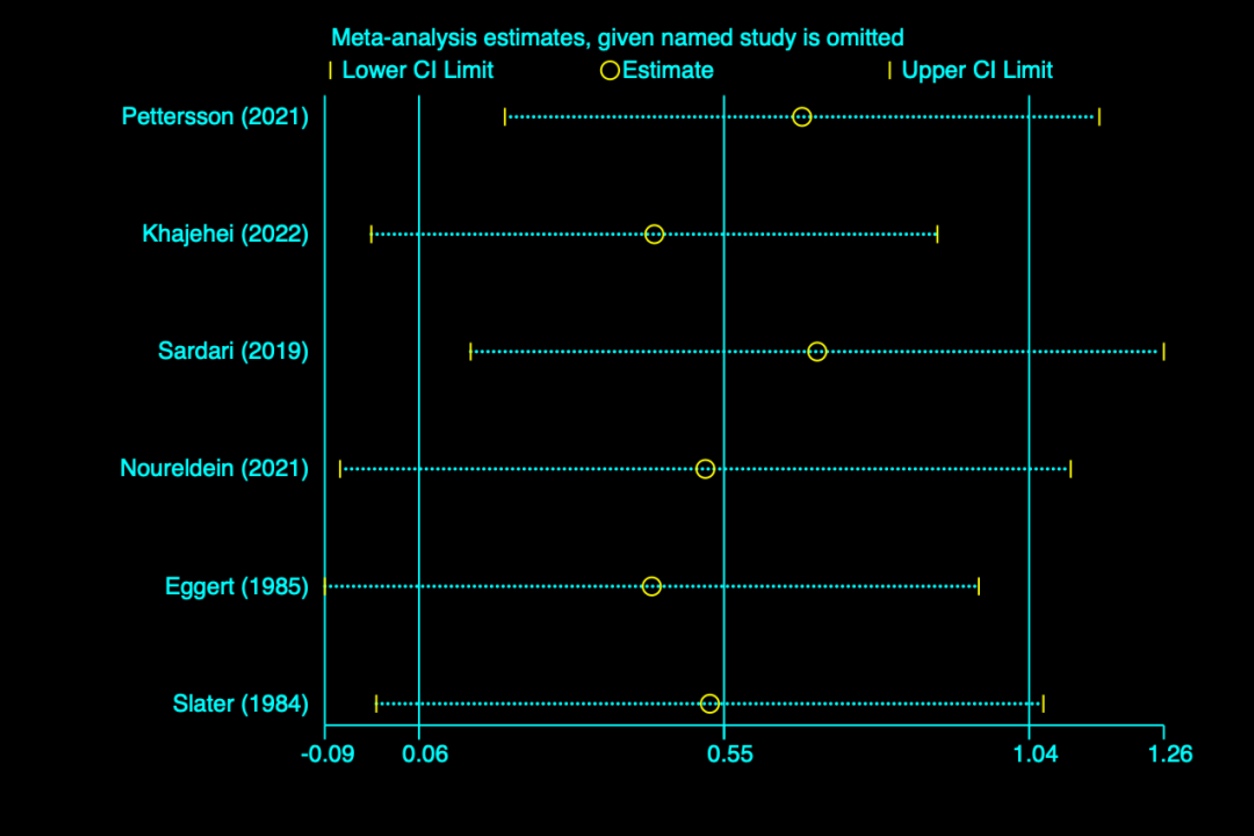


**Additional File S10-B Sensitivity analysis of the hospital readmission for HPT VS IPT**


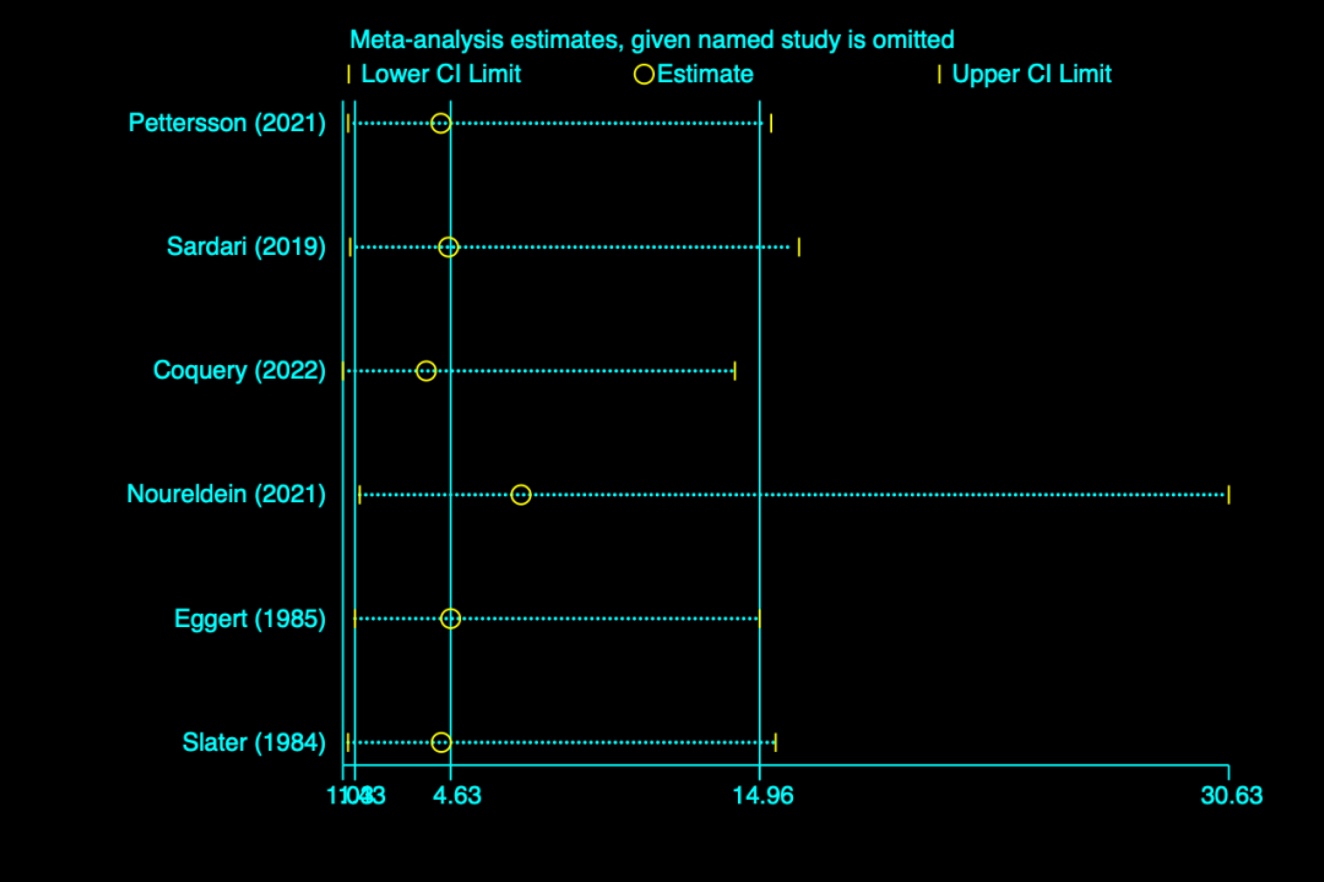


**Additional File S11. Funnel plot**

**Additional File S11-A Funnel plot of the duration of phototherapy for HPT VS IPT**


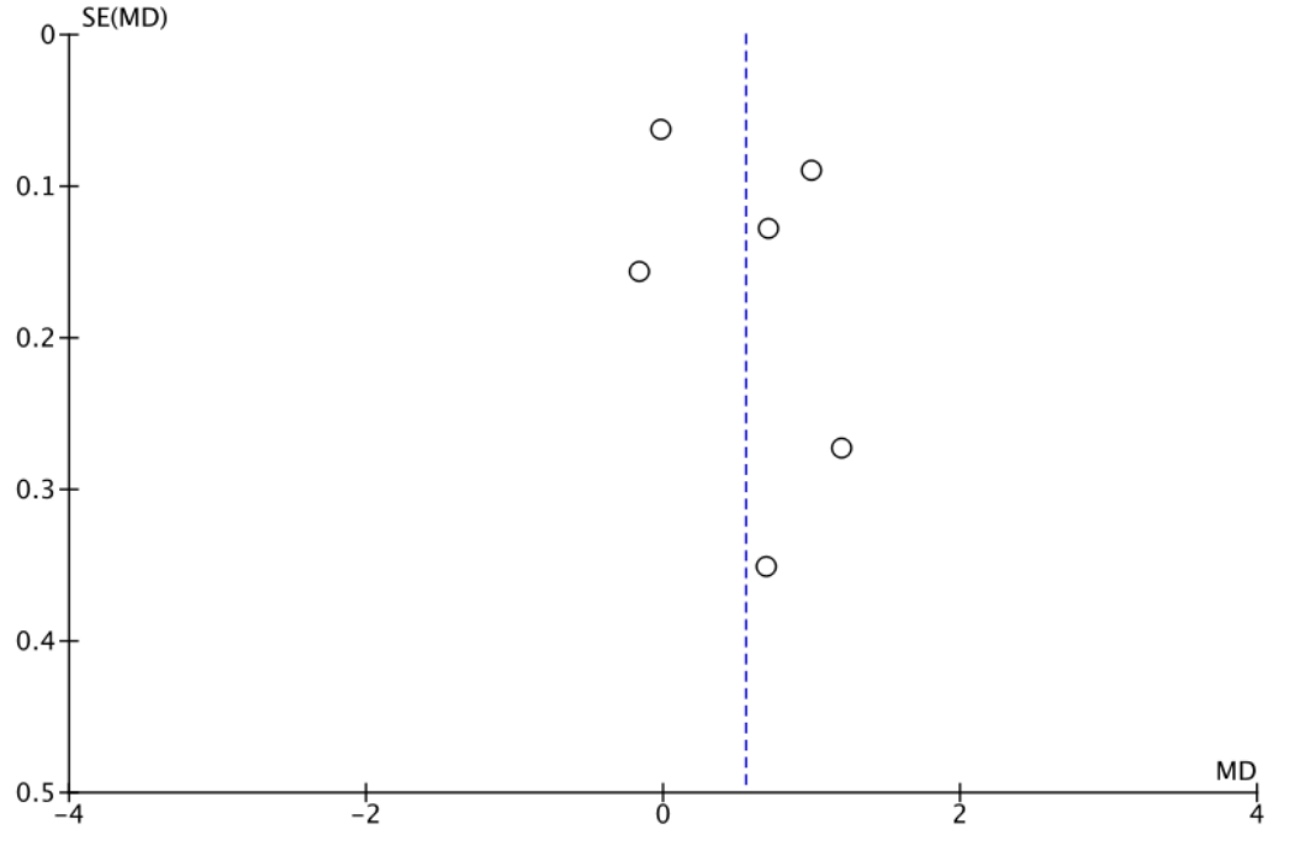


**Appendix S11-B Funnel plot of the hospital readmission for HPT VS IPT**


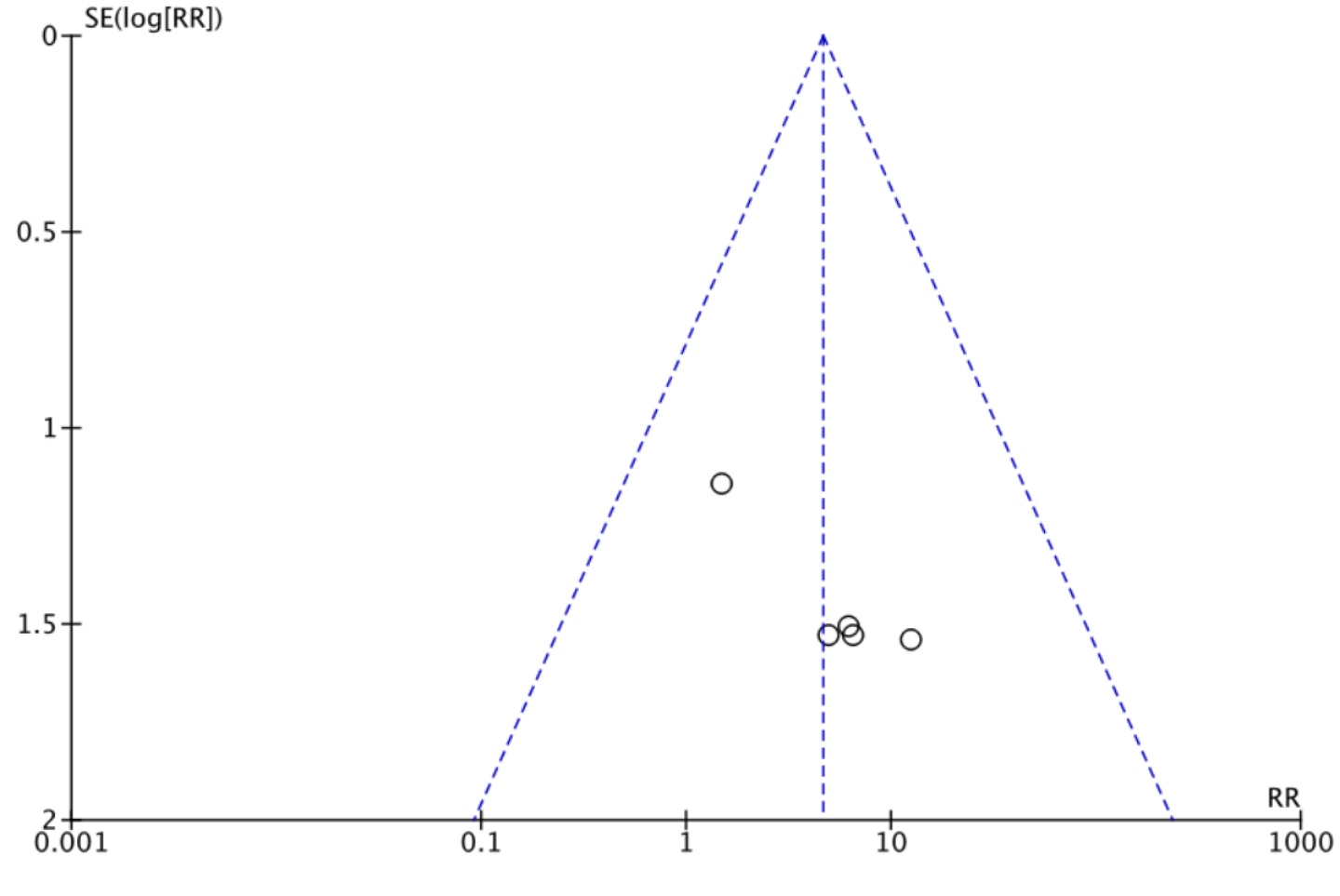

Supplement: Supplementary file 1 — Additional file 1: Additional file S1. Search strategies. Additional file S2. Eligibility criteria during selection. Additional file S3. Criteria for grading methodological quality. Additional file S4. Basis for grouping in sub-analysis. Additional file S5. List of references with final exclusion reasons. Additional file S6. Study inclusion criteria and guidelines. Additional file S7. Cochrane RoB to rate the risk of bias in RCTs. Additional file S8. The NOS to rate the risk of bias in cohort and case-control studies. Additional file S9. Evidence profiles. Additional file S10. Sensitivity analysis. Additional file S11. Funnel plot. [file 13052_2024_1613_MOESM1_ESM.docx]
